# Jaundice as a Rare Manifestation of Epstein-Barr Virus Primary Infection

**DOI:** 10.7759/cureus.15609

**Published:** 2021-06-12

**Authors:** Lígia Rodrigues Santos, Margarida Silva Cruz, Rita Veiga Ferraz, Vera Ferraz Moreira, Alice Castro

**Affiliations:** 1 Internal Medicine, Centro Hospitalar do Tâmega e Sousa, Penafiel, PRT; 2 Infectious Diseases, Centro Hospitalar do Tâmega e Sousa, Penafiel, PRT

**Keywords:** cholestasis, epstein–barr virus, hepatitis, infectious mononucleosis, jaundice

## Abstract

Epstein-Barr virus (EBV) primary infection usually presents with classic symptoms of infectious mononucleosis (IM) like fever, lymphadenopathies and tonsillopharyngitis. Liver damage is frequently mild and self-limited and there are only a few cases of severe EBV-induced cholestatic hepatitis and jaundice reported in the literature. The authors present the case of a 22-year-old woman who was admitted with acute fever and jaundice. Physical examination revealed posterior cervical lymphadenopathies and painful hepatosplenomegaly. Laboratorial findings suggested an obstructive cause for jaundice but ultrasound and magnetic resonance cholangiopancreatography excluded biliary duct pathology. Heterophile antibodies were negative but EBV-specific antibodies revealed isolated positive viral capsid antigen (VCA) immunoglobin (Ig) M suggesting the diagnosis of early phase of EBV primary infection. The diagnosis of EBV-induced cholestatic hepatitis was confirmed after identification of EBV deoxyribonucleic acid (DNA) in blood and by liver biopsy. Supportive management was provided and, despite an initial clinical deterioration, the patient had a favorable outcome. EBV is a virus with a high prevalence worldwide, mainly subclinical, and jaundice is a rare manifestation of the infection. Although the majority of the patients recover without sequelae, progression to liver failure has been described and a careful assessment for complications is mandatory. Therefore, EBV infection should be included in the comprehensive differential diagnosis of jaundice in all age groups.

## Introduction

Epstein-Barr virus (EBV) is a common herpesvirus with usually asymptomatic primary infection, occurring between 10 and 30 years old in developed countries [1, 2]. Increasing age seems to enhance the likelihood of symptomatic infection. Classic presentation as infectious mononucleosis (IM) includes fever, tonsillopharyngitis and lymphadenopathies [1, 2].

However, EBV can hypothetically affect any organ, with hematologic and liver abnormalities occurring in a large number of patients. Up to 80-90% of the patients have transaminases elevation, which is frequently mild, asymptomatic and self-limited, however, severe hepatitis and liver failure have been described especially in immunosuppressed patients [3-5]. Significant cholestasis and jaundice are rare with an estimated incidence below 5% [4-7]. Only a few cases of EBV-induced cholestasis have been reported, with or without associated features of IM [8-14].

## Case presentation

A 22-year-old woman presented in the Emergency Department with fever, abdominal pain, jaundice and choluria. No nausea, vomiting, gastrointestinal changes or other associated symptoms were reported. Three days before presentation a self-limited upper respiratory tract infection with throat pain, hoarseness and nasal congestion was mentioned. There was no history of illicit drug use, recent travel, contact with ill persons, hepatotoxic products consumption or other relevant epidemiological factors. Past personal or familiar medical history was unremarkable.

On physical examination the patient was febrile, hemodynamically stable, had skin and scleral icterus, pericentimetric posterior cervical lymphadenopathies and painful hepatosplenomegaly. Initial laboratorial tests revealed lymphomonocytosis (60% lymphocytes and 17% monocytes), mild thrombocytopenia, inflammatory markers elevation and direct hyperbilirubinemia with cytocholestasis (Table 1). Albumin and coagulation tests were within normal values. Urine tested positive for bilirubin and urobilinogen. Paul Bunnell Davidson test was negative.

**Table 1:** Baseline laboratory tests ALP, alkaline phosphatase; ALT, alanine transaminase; AST, aspartate transaminase; CRP, C-reactive protein; ESR, erythrocyte sedimentation rate; GGT, gamma-glutamyl transpeptidase; LDH, lactate dehydrogenase; WBC, white blood cells.

Laboratory findings	Baseline	Reference range
Hemoglobin (g/dl)	13.4	12-15
WBC (counts/µL)	9610	4500-11,000
Lymphocytes (counts/µL)	5766	1500-4000
Monocytes (counts/µL)	1634	200-800
Platelet (counts/µL)	138,000	150,000-400,000
ESR (mm/h)	38	0-20
CRP (mg/L)	175	<5
Total bilirubin (mg/dL)	6.6	<1.0
Direct bilirubin (mg/dL)	4.7	<0.2
ALP (U/L)	498	34-104
GGT (U/L)	78	<32
ALT (U/L)	105	<31
AST (U/L)	167	<31
LDH (U/L)	2327	266-500

**Figure 1:**
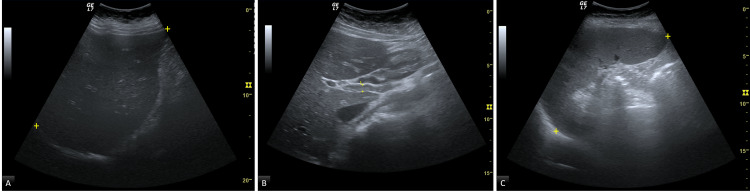
Abdominal ultrasonography A: Enlarged liver, 191mm, without focal lesions. B: Common biliary duct dilatation, 6mm, without cholelithiasis. C: Splenomegaly, 154mm. Structures marked between the yellow crosses.

Abdominal ultrasonography revealed heterogenous enlarged liver, mild common biliary duct dilatation without cholelithiasis and homogeneous splenomegaly (Figure 1). Portal vein thrombosis was excluded on doppler examination.

In the following days after admission, a clinical worsening was observed with fatigue, persistent fever and diffuse itchy maculopapular rash including palms and plants. Cholestatic enzymes continued to rise with peak on the 10th day (Table 2).

**Table 2:** Evolution of liver biochemistry tests during the first days ALP, alkaline phosphatase; ALT, alanine transaminase; AST, aspartate transaminase; GGT, gamma-glutamyl transpeptidase.

Laboratory findings	Day 1	Day 7	Day 10	Reference range
Total bilirubin (mg/dL)	8.6	12.8	13.0	<1.0
Direct bilirubin (mg/dL)	5.3	8.1	8.1	<0.2
ALP (U/L)	528	845	1439	34-104
GGT (U/L)	85	203	427	<32
ALT (U/L)	102	70	103	<31
AST (U/L)	172	97	120	<31

Multiple blood and urine cultures were negative. Nonimmune hemolytic anemia (hemoglobin level of 11.2g/dL, reference range (RR) 12-15g/dL; reticulocyte index above 2.5 without significant peripherical blood smear changes, low haptoglobin, negative antiglobulin tests) and coagulopathy have emerged (international normalized ratio of 1.7). There was ferritin and triglycerides elevation (>1500ng/mL, RR 20-250ng/mL; and 233mg/dL, RR <150mg/dL; respectively) and mild fibrinogen consumption (185mg/dL, RR 200-400mg/dL). Not all diagnostic criteria for hemophagocytic lymphohistiocytosis (HLH) were fulfilled.

Further laboratorial investigation revealed a positive EBV viral capsid antigen (VCA) immunoglobin (Ig) M; VCA and Epstein-Barr nuclear antigen (EBNA) IgG were both negative. Serologic tests for hepatitis A, B or C viruses, cytomegalovirus (CMV), toxoplasma and human immunodeficiency virus (HIV) were negative for recent or active infection. Antinuclear, antineutrophil cytoplasmic, anti-smooth muscle, antimitochondrial and anti-liver-kidney microsomal antibodies were also negative. Thoracic and abdominal computerized tomography scan revealed pericentimetric axillary, retroperitoneal, inguinal and iliac lymphadenopathies with no other lesions. Magnetic resonance cholangiopancreatography was unremarkable.

**Figure 2:**
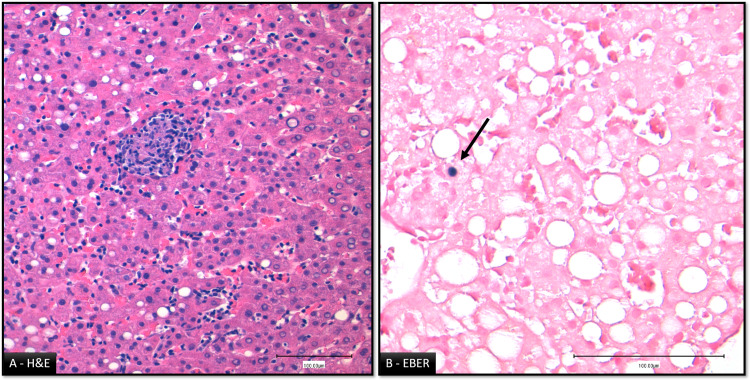
Liver histopathologic findings A: Liver parenchyma with mild steatosis, histiocytic aggregates outlining epithelioid granulomas and sinusoidal lymphocytosis. No hepatocellular necrosis was found. B: Immunohistochemical staining for Epstein-Barr virus (EBV) by EBV encoding region (EBER) revealed positivity of few hepatocytes (arrow).

EBV deoxyribonucleic acid (DNA) was detected by polymerase chain reaction (PCR, 3.7x105 copies/ml). Liver biopsy was performed and histopathological findings were compatible with inflammatory infiltration of the liver by epithelioid granulomas and sinusoidal lymphocytosis (Figure 2) and the diagnosis of cholestatic hepatitis due to EBV primary infection was confirmed.

The patient gradually improved under supportive treatment. Follow-up tests three months after discharge revealed normal liver tests and EBV seroconversion (Table 3).

**Table 3:** Evolution of liver biochemistry tests within follow-up ALP, alkaline phosphatase; ALT, alanine transaminase; AST, aspartate transaminase; GGT, gamma-glutamyl transpeptidase.

Laboratory findings	1st month	3rd month	Reference range
Total bilirubin (mg/dL)	3.1	0.6	<1.0
Direct bilirubin (mg/dL)	1.5	-	<0.2
ALP (U/L)	129	87	34-104
GGT (U/L)	33	18	<32
ALT (U/L)	64	28	<31
AST (U/L)	45	23	<31

## Discussion

Jaundice is a rare presentation of EBV primary infection. Investigation of an obstructive jaundice is mainly based on abdominal imaging, as differential diagnosis includes extrahepatic cholestasis. After its exclusion by ultrasonography, or more effectively by magnetic resonance cholangiopancreatography, early recognition of the potential causes of intrahepatic cholestasis injury, in which EBV must be considered, is essential to a correct diagnosis and management [10].

In the reported case, heterophile antibodies were negative. Despite its valuable use for point-of-care diagnosis, false-negative results can occur, especially at the initial clinical presentation [2]. EBV specific IgM antibodies were positive, documenting primary infection. Other causes were excluded, namely other viruses, drugs, autoimmune and infiltrative diseases [15].

At admission, the patient had an obstructive jaundice with direct hyperbilirubinemia and choluria. Posteriorly, we might assume that the virus-induced nonimmune hemolytic anemia contributed to jaundice as the hyperbilirubinemia became mixed. Cutaneous rash can be associated with EBV infection in 5% of patients, especially when antibiotics are prescribed [14, 16]. In this report, no antibiotic therapy has been used prior to maculopapular erythema installation.

The diagnosis of HLH was considered owing the severe documented immune activation. This aggressive and life-threatening entity should be promptly recognized and treated, as diagnosis delay can be associated with poor outcome. However, only four of the criteria were fulfilled (diagnosis is made if five of the following findings are present: fever ≥38.5°C, splenomegaly, significant peripheral blood cytopenia, hypertriglyceridemia or/and hypofibrinogenemia, hemophagocytosis evidence, low natural killer cell activity, high ferritin level and soluble CD25; or in the presence of heterozygosity for HLH-associated mutations with concurrent clinical features) [17].

The pathogenesis of cholestasis in the setting of EBV primary infection is not completely understood and several mechanisms have been proposed. It is thought that EBV infection leads to cytokine increase with inflammation and disruption of canalicular function or to direct damage of hepatic cells by autoantibody-mediated oxidative damage [3, 6, 18]. Once suspected, a detailed medical history and physical examination should be performed, and serologic markers should be measured [3, 8, 9].

## Conclusions

The final diagnosis of EBV-related jaundice requires a high level of suspicion hence the importance of reporting this rare complication of EBV infection. The management of EBV infection is mainly supportive and prognosis is usually favorable with complete recovery in several weeks. However, progression to liver failure has been described and a careful assessment should be made to prevent that.
